# Lack of sufficiently strong informative features limits the potential of gene expression analysis as predictive tool for many clinical classification problems

**DOI:** 10.1186/1471-2105-12-463

**Published:** 2011-12-01

**Authors:** Kenneth R Hess, Caimiao Wei, Yuan Qi, Takayuki Iwamoto, W Fraser Symmans, Lajos Pusztai

**Affiliations:** 1 Department of Biostatistics, University of Texas MD Anderson Cancer Center, Houston, Texas, USA; 2 Breast Medical Oncology, University of Texas MD Anderson Cancer Center Houston, Texas, USA; 3 Pathology, University of Texas MD Anderson Cancer Center Houston, Texas, USA

## Abstract

**Background:**

Our goal was to examine how various aspects of a gene signature influence the success of developing multi-gene prediction models. We inserted gene signatures into three real data sets by altering the expression level of existing probe sets. We varied the number of probe sets perturbed (signature size), the fold increase of mean probe set expression in perturbed compared to unperturbed data (signature strength) and the number of samples perturbed. Prediction models were trained to identify which cases had been perturbed. Performance was estimated using Monte-Carlo cross validation.

**Results:**

Signature strength had the greatest influence on predictor performance. It was possible to develop almost perfect predictors with as few as 10 features if the fold difference in mean expression values were > 2 even when the spiked samples represented 10% of all samples. We also assessed the gene signature set size and strength for 9 real clinical prediction problems in six different breast cancer data sets.

**Conclusions:**

We found sufficiently large and strong predictive signatures only for distinguishing ER-positive from ER-negative cancers, there were no strong signatures for more subtle prediction problems. Current statistical methods efficiently identify highly informative features in gene expression data if such features exist and accurate models can be built with as few as 10 highly informative features. Features can be considered highly informative if at least 2-fold expression difference exists between comparison groups but such features do not appear to be common for many clinically relevant prediction problems in human data sets.

## Background

Gene expression data are commonly used to develop multi-gene prediction models for various clinical classification problems. Several gene expression-based multivariate prognostic and treatment sensitivity predictors have been developed for breast cancer and numerous other "gene signatures" have been reported to predict specific biological states including pathway activity and mutation status of p53, BRCA, PIK3 and other genes in cancer [1-9]. However, many genomic predictors yielded low accuracy in independent validation [10-14]. It also seems apparent that some classification problems are easier to solve than others in the mRNA expression space. For example, it is straightforward to construct accurate classifiers for breast cancer that predict estrogen-receptor (ER) status or histologic grade due to the large scale gene expression differences that exist between ER-positive and -negative or low grade versus high grade cancers [14-17]. Many of the empirically developed first generation prognostic and predictive gene signatures for breast cancer derive their predictive value from recognizing molecular equivalents of ER status and tumor grade. This is because prognosis, drug response rates and even p53, PI3K or BRCA mutation status are not evenly distributed between ER-positive and -negative breast cancer [18]. When clinically more homogeneous subtypes of breast cancers are analyzed, it has been difficult to develop outcome predictors with good performance metrics [19].

Supervised classification models are developed through comparison of groups of samples that differ in clinical outcome of interest. The first step typically involves identification of informative probe sets/genes (i.e. features) that are differentially expressed between the groups. Next, these informative features are considered as variables to train a multivariate classification model. Intuitively, the predictive performance of classifiers must depend on the number of informative features, the magnitude of difference in feature expression levels between the groups of interest, and the number of informative cases in each group. These critical parameters are expected to vary from classification problem to classification problem and from data set to data set. However, it is not well understood how each of these components influence the success of the classifier development process and what the minimum requirement to develop successful predictors might be.

The goal of this analysis was to take public breast cancer gene expression datasets, spike these with a series of artificial "gene signatures" and assess how well these spiked-in gene signatures could be recovered and used to develop a multi-gene classifier to predict "spiked-in" status of a sample. The artificial gene signatures consisted of real probe sets whose expression values were increased (i.e. spiked) with a constant. The extent of perturbation varied over a broad range for three key parameters: (i) the number of samples perturbed (i.e. informative cases), (ii) the number of probe sets included in the artificial signature (i.e. signature size), and (iii) the fold increase in mean expression value for the spiked probes (i.e. signature strength). To place our findings into context, we also calculated gene signature size and strength for nine different real-life clinical prediction problems in six different data sets.

## Methods

### Data sets

We used 3 publically available human breast cancer gene expression data sets each generated with Affymetrix U133A gene chips. These included the Microarray Quality Control Consortium (MAQC II) breast cancer data (n = 233, Gene Expression Omnibus [GEO accession number GSE 16716] [20], the TRANSBIG data set [n = 199, GSE 7390] [3] and the Wang et al data set [n = 286, GSE 2034] [2]. Each data set was analyzed separately using identical analysis plan to assess consistency of findings. The individual Affymetrix CEL files were MAS5 normalized to a median target array intensity of 600 and expression values were transformed to log base 2 values using the Bioconductor software http://www.bioconductor.org.

### Perturbing of probe set expression values

We randomly selected *s *samples (*s *= 10, 15, 20, 25, 30, 40, 60, 80, 100) to be perturbed in each data set. In the classification exercise described below, these *s *perturbed samples represent one class and the remaining samples in the dataset represent the other class. For each *s *sample set, we randomly selected *g *probe sets (*g *= 10, 15, 20, 25, 30, 50, 100, 250, and 500) to represent the informative features (i.e. spiked gene signature). We altered the normalized, log_2_-transformed expression values of each *g *probe sets by adding the same *c *constant (*c *= 0, 0.5, 1, 1.2, 1.5, 2, 3, 4). This is equivalent to multiplying the original scale value by 2^c^. So, c = 0 corresponds to unperturbed data, c = 1.0 corresponds to a 2-fold increase and *c *= 2 corresponds to a 4-fold increase. By perturbing probe sets only in the *s *randomly selected samples, we are generating gene signatures between the two classes of increasing size and strength. We also created perturbed data sets where the probe sets were perturbed with randomly picked constants within brackets of *c *values using uniform distribution including *c *= (0.0 to 0.5), (0.50 to 1.0), (1.00 to 1.2), (1.2 to 1.5), (1.5 to 2.0), (2.0 to 2.5), (2.5 to 3.0), and (3.0 to 4.0). We repeated the entire perturbation process 20 times for each possible *s*(sample)*-g*(probe set)*-c*(constant) combination in all 3 data sets.

### Classifier model building

We used t-tests to compare the spiked cases with the rest of the cases to identify differentially expressed probe sets among all probe sets represented on the arrays. We ranked all probe sets by p-value and used the top *n *features (*n *= 10, 25, 50, 100 and 500) to construct multivariate prediction models using Diagonal Linear Discriminant Analysis (DLDA) for each *s-g-c *combination [4,19,21]. During model building we used the known perturbation status of the samples to train the models.

We tracked how many of the spiked probe sets (i.e. truly informative features) were included in the top *n *features in each iteration of the t-test. As reference point for these observations, we also used the same method to identify informative probe sets for 9 real clinical classification problems. First we compared ER-positive with ER-negative breast cancers, then cancers with pathologic complete response (pCR) to chemotherapy versus lesser response (RD), and subsequently cases with pCR versus RD among ER-negative cancers (GEO data set: GSE 16716). During these analyses we kept the overall sample sizes and the proportion of informative cases identical for each 3 comparisons in order to standardize the power of the analysis. The smallest sample size was the comparison of pCR versus RD among ER-negative cancers that included 91 cases (pCR = 41, RD = 50). Therefore, we only used a randomly sampled subset of 91 cases from the total of 233 MAQCII cases for the "ER-positive versus ER-negative" and "pCR versus RD" comparisons and also fixed the proportion of informative cases to 41 versus 50 for each comparisons. To obtain further reference values to interpret our spiked-in simulation results we also generated lists of informative probe sets to distinguish cancers that relapsed with those that did not relapse (GSE 7390, GSE 2034 and GSE11121), inflammatory from non-inflammatory breast cancer (GSE 22597), p53-mutant from p53-normal (GSE 3494) and PIK3CA-mutant from PIK3-normal cancers [9]. These analyses were performed separately for ER-positive and ER-negative cancers in each data set in order to avoid contamination by phenotype-related genes. False discovery rates were estimated using the *fdrtool *software package [22].

### Assessment of prediction models

Our goal was to predict which samples were perturbed. We built prediction models with the top 10, 25, 50, 100, 500 features selected by t-test and ranked by p-value within each *s-g-c *combination (N = 9(*s*) × 9(*g*) × 8(*c*) × 20 (replicates) = 12, 960 data sets). To estimate predictive performance, we performed stratified 3-fold Monte Carlo Cross-Validation (MCCV) with 100 iterations. During cross validation, we randomly selected 2/3 of the data to train the model and the remaining samples were used as a test set. This selection process was done separately within the spiked and non-spiked groups in order to maintain the same proportion of perturbed and unperturbed cases in the training and testing sets. New feature selection was performed during each iteration.

The classification process was assessed by two metrics: (i) the area above the receiver operating characteristic curve (AAC) which is the complement of the area under the curve (AUC) (AAC = 1-AUC) and (ii) the spiked probe recovery rate. When the total number of spiked probe sets is less than the number of features included in the classifier, we computed the recovery rate by dividing the number of correctly selected spiked probes included in the model by the number of all spiked probes. If the number of spiked probes was greater than the number of features included in the classifier we calculated the recovery rate by dividing the number of correctly selected spiked probe sets by the number of all features included in the classifier. The ability to include the perturbed/spiked probe sets in the classifier is critical for producing a model with good predictive accuracy (i.e. performance). Thus the rate at which spiked probe sets are included in the classifier (i.e., "recovered") is a useful metric for evaluating the classification process.

An R-code package has been developed to run the complete probe set spiking, model building and predictor evaluation process and is included in Additional File 1.

## Results

### The effects of sample size and signature strength on spiked-in gene recovery

**Figure 1:**
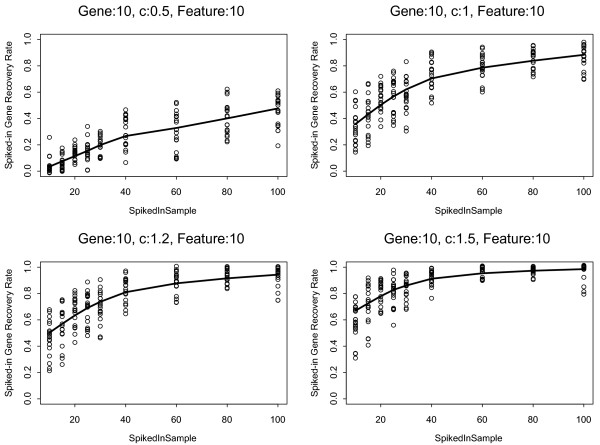
**Spiked probe set recovery rates as function of informative samples size and signature strength**. Both, the number of spiked probes (genes) and the number of features included in the predictor model were set to 10. The solid lines indicate the average recovery rates and the dots represent results from the 20 individual iterations. Results from the MAQC-II data set (n = 233) are shown. The "c" value which is the log_2 _fold-change takes on the values 0.5, 1.0, 1.2 and 1.5.

First, we examined the spiked probe set recovery rates as function of signature strength and number of spiked samples. Figure 1 shows the probe set recovery rates when the number of spiked probes was set to 10 while we varied the number of spiked-in samples from *s *= 10 to 100 (4.3% - 43% of all samples, MAQC-II data set n = 233) and the fold change of the spiked probe sets over *c *= 0.5, 1.0, 1.2, and 1.5. Fold change (i.e. signature strength) had the greatest effect on feature recovery rate. With c = 1.5, the average recovery rate was > 60% (range: 30-80%) even when only 10 samples were spiked. When the spiked sample size reached 40 (17% of all samples) the average recovery rate increased to > 80% (range: 70-100%). On the other hand, when the c was a modest 0.5, the recovery rate remained low, around 40% even when the spiked-in sample size was 100. Similar trends were seen when 50 or 500 probe sets were spiked (Supplementary Results). These observations were consistent across all 3 data sets and indicate that fold increase in the expression value of informative probe sets has a major influence on feature recovery rate. Probe sets with less than 1.5-fold difference in mean expression values between comparison groups are difficult to identify as informative features.

### The effects of sample size and signature strength on classifier performance

**Figure 2:**
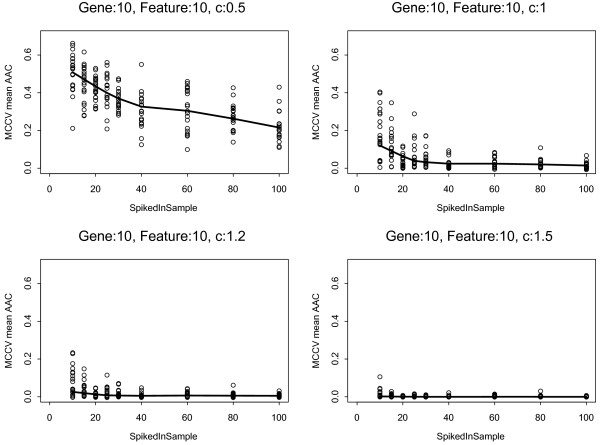
**Classifier performance as function of informative sample size and signature strength**. Both, the number of spiked probes and the number features included in the predictor model were set to 10. The solid lines indicate the average area above the ROC curve (AAC) from Monte Carlo Cross Validation (MCCV). The smaller the AAC the more accurate the predictor is. The dots represent results from the 20 individual iterations of the analysis performed on the MAQC-II data set (n = 233). The "c" value which is the log_2 _fold-change takes on the values 0.5, 1.0, 1.2 and 1.5.

Next, we examined how increasing the number of perturbed cases and the fold difference in spiked probe sets influence predictor performance. The classifier performance improved dramatically as the signature strength increased. The predictors reached almost perfect accuracy at c = 1.0 (2-fold change) when 20-25 samples (9-11% of samples) were spiked (Figure 2). Increasing the number of spiked cases gradually improved model performance when the c was a modest 0.5, but even at sample size of 100 (43% of all samples), the AAC was 0.2. The same was observed in all 3 data sets and over a broad range of spiked probe sets (*g *= 10-500), results were least sensitive to the number of features included in the classification models (Additional File 2).

We also examined the more realistic scenario when we altered the expression of the selected probe sets over a range of fold change that differed from probe set to probe set but remained within brackets of pre-specified maximum *c *including (0.0-0.5), (0.50-1.0), (1.00-1.2) and (1.20-1.5). The individual *c *constants that were added to the log2 expression values of each of the probe sets selected for spiking were randomly picked from all possible values within a given bracket. This experiment yielded similar results as presented on Figure 2 but with slightly reduced performance at signature strength below 2-fold expression change (Supplementary Results). Essentially perfect predictors could be constructed once the spiked-in sample size reached 10% of the study population and the signature strength bracket was ≥ 1.0-1.2.

### Effect of signature size on prediction accuracy

**Figure 3:**
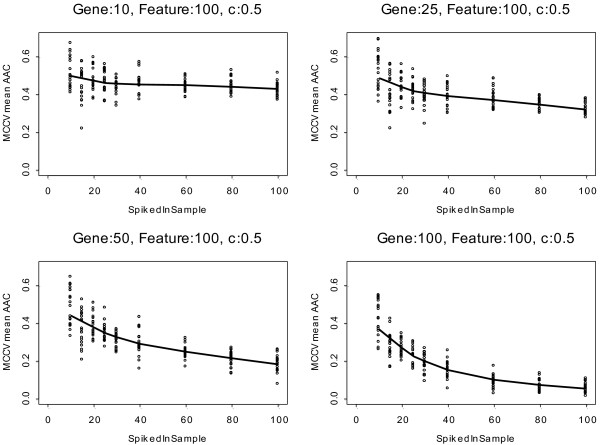
**Classifier performance is influenced by signature size, the number of informative cases and the number of features included in the prediction model**. The numbers of spiked probes were 10, 25, 50 and 100 and the number of features included in the prediction model was set to 100. Log_2 _fold-change ("c") was set to 0.5. The solid lines indicate the average area above the ROC curve (AAC) from Monte Carlo Cross Validation (MCCV), the dots represent results from the 20 individual iterations of the analysis performed on the MAQC-II data set (n = 233).

We also examined how the signature size, the number of perturbed probe sets, affects prediction performance. Since predictive accuracy rapidly reached a plateau when the fold increase in the expression of informative probe sets reached 2 (i.e., when c reached 1) (Figure 2), for this analysis we fixed c at 0.5. We varied the number of samples that were perturbed (s = 10-100) and the number of spiked probe sets from 10 to 100 but kept the feature set size used for model building at 100. Predictive performance improved gradually as the signature size increased at each sample size level (Figure 3). Predictive performance also improved as the sample size of perturbed cases increased. However, increasing the feature set size in a model led to modestly deteriorating performance when the number of informative probe sets was small and the signature strength was low, due to inclusion of noise in the model. As the number of truly informative probe sets converges towards the number of features included in the model, the performance improves rapidly even at small sample sizes.

**Figure 4:**
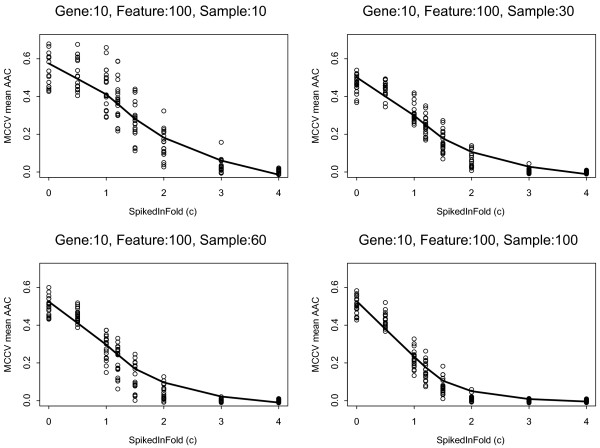
**Classifier performance as function of signature strength and informative sample size when redundant features are also included in the model**. The number of spiked probes was set to 10 and the number of features included in the predictor was set to 100. The informative sample sizes were 10, 30, 60, 100 and the log_2 _fold increases (i.e., c values) were, 0.5, 1.0, 1.2, 1.5, 2.0, 3.0, 4.0. The solid lines indicate the average area above the ROC curve (AAC) from Monte Carlo Cross Validation (MCCV), the dots represent results from 20 iterations of the analysis performed on the MAQC-II data set (n = 233).

To further explore the effect of noise (i.e. uninformative features included in the model) on predictor performance, we kept the number of spiked probes at 10 and set the the predictive model size at 100 features. Figure 4 shows that increasing the signature strength dramatically improves predictive performance even if a large number of non-informative features are included in the model. For example, if a gene signature consists of 10 probe sets each of which has a 4-fold increase in expression in a subset of 4.3% (n = 10) of the entire study population a highly accurate predictor could be built with an AAC of 0.2. These results indicate that the most critical determinant of model performance is signature strength. If the magnitude of expression difference is ≤ 2 fold and the true signature size is small, model performance depended strongly on the number of cases that were perturbed and on the number of non-informative probe set included in the model. Greater than 30% of samples need to be informative in order to develop and train a model with an AAC around 0.2-0.3 if the true signature includes only 10 probe sets with a 2-3 fold difference but also includes a large number of spuriously selected, non-informative features (90 of 100).

### Gene signature size and strength for real clinical prediction problems

Genomic classifiers developed to predict clinical outcome in breast cancer usually yield substantially lower AUC values than what we could achieve in our simulation experiments [13,19,20]. This suggests that real life clinical classification problems often involve low level expression differences in a modest number of genes (i.e. < 2 fold difference in < 10 genes). To estimate signature size and strengths for various real-life prediction problems we calculated fold change differences for the top 100 most differentially expressed probe sets between (i) ER-positive and ER-negative cancers, (ii) highly chemotherapy sensitive (i.e. those who achieved pCR) and less sensitive cancers and (iii) chemotherapy sensitive versus less sensitive cancers among ER-negative breast cancers using a standardized sample size of 91 and a fixed proportion of informative cases for each of these comparisons. In the ER-positive versus -negative comparison, the top 10 probe sets all had > 2 fold mean expression difference with very low FDR. In the pCR versus lesser response comparison without stratification by ER status, the top 10 list included only 3 genes that had > 2 fold difference but all the rest had expression difference between 1-2 fold and all features had low FDR. When the same analysis was restricted to ER-negative patient only, the top 10 differentially expressed list contained no genes with ≥ 1.2 fold expression difference and FDR values were high suggesting that many of these may not be truly informative. Even after extending the differentially expressed list to include the top 100, it contained only 2 probe sets whose expression difference was ≥ 1.2 and < 2 (Table 1). We found similarly small and weakly informative signatures for 6 other prediction problems and the associated FDR values were high (Table 2).

**Table 1:** Fold difference in the expression values of informative probe sets for 3 different clinical prediction problems assessed in the same breast cancer data set (GEO GSE 16716)

	**ER+ versus ER-^1^**		**pCR versus RD^2^**		**pCR versus RD in ER- cancers only^3^**	
	Feature # 10	Feature # 100	Feature # 10	Feature # 100	Feature # 10	Feature # 100
FDR adjusted p-value	4.71E-12	3.26E-07	0.004	0.0205	0.4	0.68
Fold difference^4^						
< 0.5	0	0	0	6	4	59
≥0.5 - < 1.0	0	13	3	43	2	32
≥1.0 - < 1.2	0	**8**	**1**	**15**	**4**	**7**
≥1.2 - < 1.5	0	**18**	**2**	**17**	0	**1**
≥1.5 - < 2.0	0	**18**	**2**	**11**	0	**1**
≥ 2.0 - < 3.0	**6**	**28**	**2**	**8**	0	0
≥ 3.0 - < 4.0	**3**	**12**	0	0	0	0
≥ 4.0	**1**	**3**	0	0	0	0

**^1^**Random sample of 41 ER Positive and 50 ER Negative Samples

**^2^**Random sample of 41 pathologic CR and 50 residual cancers regardless of ER status.

**^3^**Random sample of 41 pathologic CR and 50 residual cancers all ER Negative.

**^4^**Log_2 _Difference = abs(mean log_2 _intensity for group1 - mean log_2 _intensity for group 2) where "abs" is absolute value.

The numbers of probe sets with a given level of differential expression are shown for the 3 comparisons including (i) Estrogen Receptor (ER)-positive versus ER-negative cancers, (ii) cancers with pathologic complete response (pCR) to chemotherapy versus lesser response (RD) and (iii) cases with pCR versus RD in ER-negative cancers only. Probe sets with mean log_2 _transformed expression difference **>**1 between comparisons groups are highlighted in bold. FDR = false discovery rate (i.e., proportion of genes detected to be informative which are not truly informative). FDR adjusted p-values (also known as FDR q-values) are the estimated FDR values that would be incurred if the p-values associated with the selected genes were used as the threshold for significance (i.e., genes with that p-value or a lower p-value were to be detected as informative). So for the Feature #10 column, the reported FDR adjusted p-value is the q-value associated with the 10th highest ranked gene.

**Table 2:** Fold difference in the expression values of informative probe sets for 6 different prediction problems in different data sets.

Data set	IBC versus Non-IBC				p53 mutation versus normal				PIK3 mutation versus normal			
Receptor status	ER-negative		ER-positive		ER-negative		ER-positive		ER-negative		ER-positive	
Phenotype	IBC	Non-IBC	IBC	Non-IBC	mutation	normal	mutation	normal	mutation	normal	mutation	normal
Pts #	19 vs 27		6 vs 31		44 vs 11		14 vs 31		8 vs 49		15 vs 57	
Feature #	10	100	10	100	10	100	10	100	10	100	10	100
FDR q value	1.000	1.000	0.280	0.500	0.14	0.33	0.49	0.6	0.08	0.09	0.24	0.55
Fold difference *												
< 0.5	0	20	0	2	1	9	2	29	0	0	0	17
≥0.5 - < 1.0	4	55	1	41	1	37	5	47	1	23	6	50
≥1.0 - < 1.2	**2**	**11**	**1**	**10**	**1**	**11**	**1**	**11**	3	**18**	**1**	**17**
≥1.2 - < 1.5	**3**	**12**	**4**	**18**	**2**	**15**	**1**	**9**	4	**21**	2	**10**
≥1.5 - < 2.0	**0**	**0**	**2**	**20**	2	**22**	1	**4**	1	**23**	0	**5**
≥2.0 - < 3.0	0	**1**	2	**9**	**3**	**6**	**0**	**0**	1	14	1	1
≥3.0 - < 4.0	1	**1**	0	**0**	**0**	**0**	**0**	**0**	0	1	0	0
≥4.0	0	0	0	0	**0**	**0**	**0**	**0**	0	0	0	0
Data set	WANG	TRANSBIG	Mainz									
Receptor status	ER-negative	ER-positive	ER-negative	ER-positive	ER-negative	ER-positive						
Recurrence	Yes	No	Yes	No	Yes	No	Yes	No	Yes	No	Yes	No
Pts #	29 vs 50	29 vs 50	18 vs 45	23 vs 112	11 vs 20	30 vs 139						
Feature #	10	100	10	100	10	100	10	100	10	100	10	100
FDR q value	0.2	0.26	0.004	0.02	0.820	0.91	0.010	0.03	0.47	0.58	0.01	0.03
Fold difference *												
< 0.5	2	35	3	68	2	26	1	26	1	13	3	59
≥0.5 - < 1.0	6	51	7	30	4	47	5	52	5	44	5	26
≥1.0 - < 1.2	0	**7**	**0**	**2**	**3**	**21**	**2**	**12**	0	**5**	1	**3**
≥1.2 - < 1.5	0	**5**	**0**	**0**	**1**	**6**	**1**	**8**	**1**	**16**	**0**	**0**
≥1.5 - < 2.0	**1**	**1**	**0**	**0**	**0**	**0**	**1**	**2**	**2**	**17**	**1**	**2**
≥2.0 - < 3.0	1	1	0	0	0	0	0	0	0	**3**	0	**0**
≥3.0 - < 4.0	0	0	0	0	0	0	0	0	1	2	0	0
≥4.0	**0**	**0**	0	0	0	0	0	0	**0**	**0**	**0**	**0**

*Log_2 _Difference = abs(mean log_2 _intensity for group1 - mean log_2 _intensity for group 2) where "abs" is absolute value.

The numbers of probe sets with a given level of differential expression are shown for the 6 comparisons. Analyses were performed separately for Estrogen Receptor (ER)-positive and ER-negative cancers. IBC = inflammatory breast cancer, PI3K = Phosphatidylinositol-3 kinase, FDR = false discovery rate. The WANG, TRANSBIG, Mainz data sets correspond to references 2, 3 and 28. Probe sets with mean log_2 _transformed expression difference **>**1 between comparisons groups are highlighted in bold.

These observations confirm that for easier classification problems, such as ER status prediction, a large number of informative probe sets exist and these show large fold differences but for all other prediction problems that we tested the number of informative features was low and feature strength was modest at best.

## Discussion

The goal of this project was to assess how gene signature size (i.e. the number of informative probe sets), signature strength (i.e. fold difference in the mean expression of the informative probe sets between the comparison groups) and the number of informative cases in a data set influence the success of developing multi-gene prediction models. We also examined how model performance deteriorates as increasing amount of noise (i.e. un-informative features) are included in a model. To study these variables we altered the true expression values of randomly selected genes in real human gene expression data sets. What motivated this research was to find out what signal elements in the genomic model building process may explain the substantial difficulty to find clinically relevant multi-gene predictors for many cancer classification problems.

Our results demonstrate that signature strength had the greatest influence on the success of model building followed by the size of the gene signature and the number of informative cases. Predictors tolerated large amounts of noise included in the model as long as it also contained numerous strong informative features. It was remarkably easy to develop almost perfect predictors with as few as 10 informative probe sets if the fold difference in the expression values of these probe sets was > 2.0, even if the informative samples represented < 10% of the total sample size. These simulated results are better than the reported performance of the majority of empirically developed genomic outcome predictors [10,11,23-27]. To examine the causes of this discrepancy, we calculated the number of informative probe sets and their fold difference (i.e signature strength) for 9 different real clinical prediction problems in 6 publically available breast cancer data sets. We only found sufficiently large and strong predictive signature for one prediction problem, to distinguish ER-positive from ER-negative breast cancers (87 features with ≥ 2 fold difference). Surprisingly, there was no strong signature for the clinically more relevant problems of predicting chemotherapy sensitivity among ER-negative cancers (6 of the top 10 genes had < 2 fold difference and only 9 of the top 100 genes had fold difference > 2 but all were less than 4.0 and FDR was high) and for a series of other diagnostic or mutation prediction problems.

These results indicate that current statistical methods efficiently identify highly informative features in complex gene expression data sets if such features exist. Features can be considered highly informative if at least a 2-fold expression difference exists between the means of the two comparison groups. With as few as 10 such features almost perfect classification models can be built. However, highly informative features do not appear to be common for many clinically relevant prediction problems. There may be several biological explanations for this. First, the dynamic regulation of the expression of the thousands of mRNA species in cancer cells is not well understood. What level of change in mRNA expression leads to functionally important consequences (e.g. altered chemotherapy sensitivity) is unknown and it is likely to be different from gene to gene. Some physiological variables are very tightly regulated, a 10% change in serum sodium levels can lead to life threading consequences whereas variables such as hear rate or blood pressure have broad normal dynamic range. It is likely that similar phenomena also occur in the mRNA world and a 15-50% percent change in the expression level of some mRNA species may result in functional consequences but such genes would not define strong predictive features. Also importantly, protein expression levels correlate with mRNA expression only moderately and protein levels or posttranslational changes in proteins may represent the functional activity of biological pathways more accurately than mRNA levels. Structural variations in genes at the DNA level can profoundly alter the functional activity of proteins such mutations may also not cause large scale changes in mRNA expression.

While our spike-in simulation studies yielded much useful information, they are limited in several respects. In real datasets expression values responding to regulatory influences would presumably change in a coordinated rather than random fashion. However, the degree of change and number of genes involved in a particular coordinated change may be relatively small and thus not conducive for use in a predictive model. Overall these observations are consistent with previous sensitivity analysis of prediction models that have highlighted the vulnerability of predictors to feature strength [28-30].

## Conclusions

In summary, our findings suggest that in the currently available mRNA gene expression data sets of breast cancer there may not be enough highly informative genes to develop clinically useful genomic classifies to predict certain types of clinical outcomes. We recognize that the sample sizes that are currently available for analysis are modest, and particularly small analyses are performed in disease subsets. Our simulation results suggest that predictor performance in the absence of strong predictive signatures may improve only modestly even if sample size increases. We developed an R software code package that could be used to estimate the number of informative probe sets, based on gene spiking, that is needed for accurate model building in any experimental data set. This could be used as a tool to estimate the adequacy of a given data set to yield empirically derived classifiers.

## Authors' contributions

Conception and design KH, LP; Acquisition of data WFS, YQ, LP; Analysis of data CW, KH, YQ, TI, LP; Interpretation of data KH, CW, YQ, TI, WFS, LP; Drafting the manuscript or revising KH, CW, YQ, TI, WFS, LP; Final approval KH, CW, YQ, TI, WFS, LP.

## Grant Support

The Breast Cancer Research Foundation (LP, WFS)

## Supplementary Material

Additional file 1 **R Code**. R language programming code used to perform the analyses presented in the paper. Click here for file

Additional file 2 **Supportive Results**. Supportive results of analyses on additional datasets. Click here for file
