# Supplementary material for: Lack of sufficiently strong informative features limits the potential of gene expression analysis as predictive tool for many clinical classification problems
Source: BMC Bioinformatics. 2011 Dec 1;12:463. doi: 10.1186/1471-2105-12-463 (PMC3245512; doi:10.1186/1471-2105-12-463)
Supplement: Additional file 1 — R Code. R language programming code used to perform the analyses presented in the paper. [file 1471-2105-12-463-S1.DOC]

Additional Files #1 (R-code)

################################################################################

## The following function performs two sample equal t-test

##

## We output the ranks of the absolute values of the t statistics;

##

################################################################################

library(ClassComparison)

## package "ClassComparison" can be downloaded from MDACC Bioinformatics website at http://bioinformatics.mdanderson.org/software.html;

features.t.uneqvar <- function(data,class)

{

mttest <- MultiTtest(data,as.factor(class))

out <- data.frame(cbind( mttest@t.statistics,mttest@p.values, mttest@t.statistics))

dimnames(out)[[1]] <- rownames(data)

out[,1] <- rank(20-abs(out[,1]))

dimnames(out)[[2]] <- c('ranks','p.value','stats')

out

}

################################################################################

## The following function performs stratified 3-fold Monte Carlo Cross-Validation (MCCV)

##

### Variable definitions

# dataset, is the original microarray data matrix where columns are the cases and rows are the probe sets of U133A

# nfold =3, is the n-fold for our stratified MCCV;

# nm is a vector of the dimensions of the original microarray data matrix where columns

# are the cases and rows are the probe sets of U133A;

# my.class is a vector of 0's and 1's to indicate classes of the whole data;

# data.train is a matrix where columns are the training cases and rows are the probe sets of U133A;

# class.train is a vector of 0's and 1's to indicate classes of the training data;

# class.test is a vector of 0's and 1's to indicate classes of the test data;

# my.train is a matrix where columns are the training cases and rows are top features selected by t-test;

# my.test is a matrix where columns are the testing cases and rows are top features selected by t-test;

#

################################################################################

my.MCCV2 <- function(dataset, nfold, my.mccvrep){

my.class <- my.perturbsample.ind

################ Split the samples into training and testing sets ##############

n.test <- floor( nm[2]/nfold)

factor <- mean(my.class == 1)

n.test.class.1 <- round(factor*n.test)

n.test.class.0 <- n.test - n.test.class.1

n.train <- nm[2] - n.test

AAC<- selected.spiked.gene <- array(0,c(length(n.feature),my.mccvrep))

for(replication in 1:my.mccvrep) { ## begin MCCV loop

samp.test <- c(sample( (1:nm[2])[my.class==1] ,n.test.class.1),sample( (1:nm[2])[my.class==0] ,n.test.class.0))

data.train <- dataset[,-samp.test]

class.train <- my.class[-samp.test]

################ Feature Selection using the training data set

out <- features.t.uneqvar(data.train,class.train)

index.feat <- out$ranks

for (i in 1:length(n.feature)){ #begin selecting top n.features

n.genes <- this.n.genes<- n.feature[i]

################ DLDA classification

index.genes <- (1:nm[1])[ index.feat <= this.n.genes ]

class.train <- my.class[-samp.test]

class.test <- my.class[samp.test]

my.train <- dataset[index.genes,-samp.test]

my.test <- dataset[index.genes,samp.test]

m.1 <- apply(my.train[,class.train==1],1,mean)

v.1 <- apply(my.train[,class.train==1],1,var)

m.0 <- apply(my.train[,class.train==0],1,mean)

v.0 <- apply(my.train[,class.train==0],1,var)

v <- ((sum(class.train==1)-1)*v.1 + (sum(class.train==0)-1)*v.0)/(length(class.train)-2)

d.1 <- t(my.test)%*%(m.1/v) - c(.5*(m.1/v)%*%m.1)

d.0 <- t(my.test)%*%(m.0/v) - c(.5*(m.0/v)%*%m.0)

res <- (d.1 > d.0) + 0

d <- d.1 - d.0

AAC[i,replication] <- round(1-as.numeric(wilcox.test(d[class.test==1], d[class.test==0])$statistic)/(n.test.class.1*n.test.class.0),3)

selected.spiked.gene [i,replication] <- sum (sample.genes %in% index.genes) #number of spiked-in genes selected into the top features.

} #end of the n.feature loop

} #end of the MCCV loop

out.mccv <- cbind(ii,my.perturbsample, my.perturbgene, my.perturbfold , n.feature, AAC, selected.spiked.gene)

out.mccv

################ End DLDA classification

} # end of my.MCCV2 function

#################################################################################

## The following code chunk is the one we use to generate the results for our manuscript for the MAQCII data set.

## It reads in our log-2 transformed gene expression data saved as an .Rdata file,

## alters of probe set expression values, and runs the MCCV to generate the results for the manuscript.

## this process take a very long time to finish. For the purpose of validating the R codes,

## please run the small example code below.

## We randomly selected s samples (s=10, 15, 20, 25, 30, 40, 60, 80, 100) to represent

## the informative "spiked-in" cases in each data set. For each s sample set, we randomly

## selected g probe sets (g = 10, 15, 20, 25, 30, 50, 100, 250, and 500) to represent the

## informative features (i.e. spiked in gene signature). We altered the expression values

## of each g probe sets by adding the same c constant (c = 0, 0.5, 1, 1.2, 1.5, 2, 3, 4)

##to the normalized log 2 transformed expression values.

###

##

### Variable definitions

# out.all is a matix which stores the output of the performance metrics, the 1st to 5th columns are

# the number of repeat, number of perturbed sample, number of perturbed genes, the constant we added to selected gene,

# the number of top features we used for our classifier, respectively. The 6th-105th columns are the AAC of the 100 MCCV

# repeats; and the 106th-250th columns are the number of spiked-in genes of the top features we selected.

#

# my.dat and MAQCII is a matrix of the original gene expression data where columns are the cases and rows are the probe sets of U133A;

# my.perturb.dat is a matrix of the perturbed expression data where columns are the cases and rows are the probe sets of U133A;

# perturbsample is a vector of the number of samples we are to select to be the informative "spiked-in" cases;

# perturbgene is a vector of the number of probe sets we are use to represent the informative features;

# perturbfold is the vector of the constants we are to add to the normalized log 2 transformed expression values

# of the informative features of the "spiked-in" cases;

# my.mccvrep is the number of MCCV repeats we will perform

# n.feature is a vector of the top features we will select to perform DLDA classification

# my.perturbsample.ind is vector of 0's and 1's to indicate the classes ("spiked-in" vs "non=spiked-in") of the whole data;

# my.perturbgene.ind is vector of 0's and 1's to indicate the "spiked-in" status of the probe sets;

################################################################################

load("Expression.Rdata" ) # Expression.Rdata contains the original MAQCII expression data named "MAQCII"

out.all <- NULL

my.dat <- MAQCII;

nm <- dim(my.dat);

perturbsample <- c ( 10, 15, 20, 25, 30, 40, 60, 80, 100)

perturbgene <- c( 10, 15, 20, 25, 30, 50, 100, 250, 500)

perturbfold <- c(0, 0.5, 1, 1.2, 1.5, 2, 3, 4 )

my.mccvrep <- 100

nfold <- 3

n.feature=c(10, 25, 50, 100, 500)

for (ii in 1: 20) { # ii is the number of repeats of s-g-c process

for (jj in 1: length(perturbsample)) {

my.perturbsample <- perturbsample[jj]

my.perturbsample.ind <- sample (rep(c(1,0),c(my.perturbsample,dim(my.dat)[[2]]- my.perturbsample)), dim(my.dat)[[2]], replace = FALSE)

for (kk in 1: length(perturbgene)) {

my.perturbgene <- perturbgene [kk]

my.perturbgene.ind <- sample (rep(c(1,0),c(my.perturbgene,dim(my.dat)[[1]]- my.perturbgene)), dim(my.dat)[[1]], replace = FALSE)

sample.genes <- (1:nm[1]) [my.perturbgene.ind==1]

my.mult <- my.perturbgene.ind %*% t(my.perturbsample.ind)

for (ll in 1:length(perturbfold)) {

my.perturbfold <- perturbfold [ll]

my.mult.per <- my.mult * my.perturbfold

my.perturb.dat <- my.dat + my.mult.per

out.mccv <- my.MCCV2(dataset=my.perturb.dat, nfold, my.mccvrep)

out.all <- rbind(out.all , out.mccv)

} ##loop of ll

} ##loop of kk

} ##loop of jj

} ##loop of ii

colnames(out.all) <- c("rep.process","s-sample", "g-genes", "c-constant","n-feature",

paste("AAC.rep",1:my.mccvrep), paste("Gene.recovery.rep",1:my.mccvrep))

#####################################################################################

# Now we can run an small example, for

## the number of repeats of s-g-c = 2

## the number of spike-in samples= 10 or 80

## the number of spike-in genes= 10 or 100

## the number of spike-in fold= 0.5 or 2

## the number of MCCV repetition=2

## the number of top features selected= 10 or 100

# users can change any of the above input values to fit their need.

# Need to first run the two functions "features.t.uneqvar", "my.MCCV2".

#####################################################################################

load("sample.Expression.Rdata" )

# sample.Expression.Rdata contains the first 2000 rows of the original MAQCII expression data named "sample.data"

out.all <- NULL

my.dat <- sample.data;

nm <- dim(my.dat);

rep.process <- 2 # the number of repeats of s-g-c

perturbsample <- c ( 10, 80)

perturbgene <- c( 10, 100)

perturbfold <- c(0.5, 2 )

my.mccvrep <- 2

nfold <- 3

n.feature=c(10, 100)

for (ii in 1: rep.process) {

for (jj in 1: length(perturbsample)) {

my.perturbsample <- perturbsample[jj]

my.perturbsample.ind <- sample (rep(c(1,0),c(my.perturbsample,dim(my.dat)[[2]]- my.perturbsample)), dim(my.dat)[[2]], replace = FALSE)

for (kk in 1: length(perturbgene)) {

my.perturbgene <- perturbgene [kk]

my.perturbgene.ind <- sample (rep(c(1,0),c(my.perturbgene,dim(my.dat)[[1]]- my.perturbgene)), dim(my.dat)[[1]], replace = FALSE)

sample.genes <- (1:nm[1]) [my.perturbgene.ind==1]

my.mult <- my.perturbgene.ind %*% t(my.perturbsample.ind)

for (ll in 1:length(perturbfold)) {

my.perturbfold <- perturbfold [ll]

my.mult.per <- my.mult * my.perturbfold

my.perturb.dat <- my.dat + my.mult.per

out.mccv <- my.MCCV2(dataset=my.perturb.dat, nfold, my.mccvrep)

out.all <- rbind(out.all , out.mccv)

} ##loop of ll

} ##loop of kk

} ##loop of jj

} ##loop of ii

colnames(out.all) <- c("rep.process","s-sample", "g-genes", "c-constant","n-feature",

paste("AAC.rep",1:my.mccvrep), paste("Gene.recovery.rep",1:my.mccvrep))

out.all
