# Supplementary material for: Lack of sufficiently strong informative features limits the potential of gene expression analysis as predictive tool for many clinical classification problems
Source: BMC Bioinformatics. 2011 Dec 1;12:463. doi: 10.1186/1471-2105-12-463 (PMC3245512; doi:10.1186/1471-2105-12-463)
Supplement: Additional file 2 — Supportive Results. Supportive results of analyses on additional datasets. [file 1471-2105-12-463-S2.DOC]

**Additional Files 2**

**(supportive results in other data sets)**

*Data sets*

We used the following 3 data sets:

1. MAQC II breast cancer data (GEO: GSE 16716): total number of samples= 233
2. TRANSBIG data set (GEO: GSE 7390): total number of samples=199
3. Wang data set (GEO: GSE 2034): total number of samples=286

*Spike-in*

We randomly select s (s=10, 15, 20, 25, 30, 40, 60, 80, 100) samples to be the "spiked-in' samples, and for the selected "spiked-in' samples, we randomly selected

g (g= 10, 15, 20, 25, 30, 50, 100, 250, 500) genes to the "spiked-in' signatures. For each s/g set, we add a Constance c (c=0, 0.5, 1, 1.2, 1.5, 2, 3, 4) to the selected "spiked-in' signatures. We repeat the whole “spiked-in” process 20 times.

*Classification Method*

We chose one well known, widely used, and widely studied classification method: Diagonal Linear Discriminant Analysis (DLDA). DLDA used linear combinations to classify samples. Each test sample is assigned the class with closest centroid of the linear combination. This makes DLDA a global method with very stable class boundaries.

*Feature Selection*

We log transformed (base 2) the normalized data and used the t-test to rank genes (i.e., probesets) for each “spiked-in” dataset. The numbers of top features we use are 10, 25, 50, 100, and 500. This spans the range from parsimonious models to highly multiplex models.

*Cross-Validation*

For each spiked-in data set, we performed stratified 3-fold Monte Carlo cross-validation (MCCV) cross validation. The stratification ensures that the ratio of cases and non-cases are roughly equal in the training and testing sets. For each MCCV split, we randomly select 2/3 of the data (selecting separately within the two classes to maintain balance, i.e. the proportion of case/normal in the training and testing sets is similar to those in the whole set) to train the model, and then test on the remaining 1/3 of the samples. Feature selection is included in the cross-validation. This step is necessary to avoid the bias due to using the same data to estimate the model parameters and to estimate performance. We repeat the MCCV 100 times for each “spiked-in” data set.

*Performance evaluation*

We report 3 outcomes: (1) the area above the ROC curve; (2) misclassification error rate; and (3) the proportion of spiked in genes recovered by our feature selection. We computed the recovery rate of the spike in genes by dividing the number of selected spiked-in genes by the number of spiked in genes if the number of spiked in genes is less or equal to the number of features included in the classifier, and by dividing the number of selected spiked-in genes by the number of features included in the classifier if the number of spiked in genes is greater than the number of features included in the classifier.

**Spiked-In Gene Experiment Supplementary Results I**

**The Wang Data Set**

Normalized data downloaded from GEO and expression values were log2-transformed and target intensity was set to 600.

We have nine values for the number of spiked-in samples (SpikeInSample): 10, 15, 20, 25, 30, 40, 60, 80, and 100; nine values for the number of spiked-in genes (SpikeInGene): 10, 15, 20, 25, 30, 50, 100, 250, and 500; eight values for the fold increase in the spiked-in genes (SpikeInFold): 0, 0.5, 1, 1.2, 1.5, 2, 3, and 4; and 5 values for the number of features included in the classifier: 10, 25, 50, 100, and, 500. Below are plots of area above the ROC (AAC).

AAC decreases as the number of samples increase. Here we vary the fold change for 10 spiked-in genes and 10 features. Here again we see floor effects for large values of fold and the number of samples. The classifiers are predicting perfectly and AAC = 0.

Here we fix the fold increase to 0.5 but vary the number of spiked-in genes. Note that the rate of decrease of AAC as a function of the number of spiked-in samples increases as the number of spiked-in genes increases.

Here we plot AAC vs. the number of spiked-in genes for 100 features and fold = 1.0. The number of spiked-in samples is increased over 10, 25, 60 and 100. AAC decreases as the number of spiked-in genes increases and this decrease becomes more pronounced as the number of spiked-in samples increases. Floor effects are seen at higher numbers of genes.

Here we plot AAC vs. the fold increase in the spiked-in genes. The number of spiked in genes is set at 10 and the number of features included in the classifier is set at 100. The number of spiked-in samples increases over: 10, 30, 60 and 100. AAC decreases as the fold of the spiked-in genes increases.

Here we plot AAC vs. the fold increase in the spiked-in genes. The number of spiked in samples is set at 100 and the number of features included in the classifier is set at 100. The number of spiked-in genes increases over: 10, 20, 30 and 50. AAC decreases as the fold of the spiked-in genes increases. Floor effects are seen at higher folds.

Lastly, we plot AAC vs. the number of features for 10 spiked-in samples and the fold of the spiked genes set to 0.5. The number of spiked-in genes increases over 50, 100, 250, and 500. AAC increases slightly as the number of features increases. Note that the x-axis is plotted on the log scale.

*Rate of Spiked-in Gene Recovery*

We first plot the recovery rate vs. the number of spiked-in samples for 10 spiked-in genes and 10 features included in the classifier. The fold-change in the spiked-genes increases over 0.5, 1.0, 1.2 and 1.5. Similar plots are seen for 50 genes and 50 features and for 500 genes with 500 features.

Here we plot the recovery rate vs. the fold increase of the spike-in genes for 10 genes and 10 features. The number of spiked in samples increases over 10, 20, 30 and 40.

A similar plot for 500 genes and 500 features is shown below. The rate of increase is steeper.

**Spiked-In Gene Experiment Supplementary Results II**

**The Transbig Data Set**

Same analysis performed as for Wang et al samples. CEL files were downloaded from GEO and the normalization was performed using MAS5 algorithm, expression values were log2-transformed and target intensity was set to 600.

AAC decreases as the number of samples increase. Here we vary the fold change for 10 spiked-in genes and 10 features. Here again we see floor effects for large values of fold and the number of samples. The classifiers are predicting perfectly and AAC = 0.

Here we fix the fold increase to 0.5 but vary the number of spiked-in genes. Note that the rate of decrease of AAC as a function of the number of spiked-in samples increases as the number of spiked-in genes increases.

Here we plot AAC vs. the number of spiked-in genes for 100 features and fold = 1.0. The number of spiked-in samples is increased over 10, 25, 60 and 100. AAC decreases as the number of spiked-in genes increases and this decrease becomes more pronounced as the number of spiked-in samples increases. Floor effects are seen at higher numbers of genes.

Here we plot AAC vs. the fold increase in the spiked-in genes. The number of spiked in genes is set at 10 and the number of features included in the classifier is set at 100. The number of spiked-in samples increases over: 10, 30, 60 and 100. AAC decreases as the fold of the spiked-in genes increases.

Here we plot AAC vs. the fold increase in the spiked-in genes. The number of spiked in samples is set at 100 and the number of features included in the classifier is set at 100. The number of spiked-in genes increases over: 10, 20, 30 and 50. AAC decreases as the fold of the spiked-in genes increases. Floor effects are seen at higher folds.

Lastly, we plot AAC vs. the number of features for 10 spiked-in samples and the fold of the spiked genes set to 0.5. The number of spiked-in genes increases over 50, 100, 250, and 500. AAC increases slightly as the number of features increases. Note that the x-axis is plotted on the log scale.

*Rate of Spiked-in Gene Recovery*

We first plot the recovery rate vs. the number of spiked-in samples for 10 spiked-in genes and 10 features included in the classifier. The fold-change in the spiked-genes increases over 0.5, 1.0, 1.2 and 1.5. Similar plots are seen for 50 genes and 50 features and for 500 genes with 500 features.

Here we plot the recovery rate vs. the fold increase of the spike-in genes for 10 genes and 10 features. The number of spiked in samples increases over 10, 20, 30 and 40.

A similar plot for 500 genes and 500 features is shown below. The rate of increase is steeper.

**Spiked-In Gene Experiment Supplementary Results III**

**MAQCII Data Set with variable constant *c***

We altered the expression of the selected probe sets over a range of fold change that differed from probe set to probe set but remind within brackets of pre-specified maximum *c* including (0.0-0.5), (0.50-1.0), (1.00-1.2) and (1.20-1.5). The individual *c* constants that were added to the log2 expression values of each of the selected 10 probe sets were randomly picked from all possible values within any given bracket.

Spiked probe set recovery rates as function of samples size of informative cases and fold increase in expression difference (fold changes are from brackets)

Classifier performance as function of sample size of informative cases and fold increase in expression difference of informative probe sets ( fold changes are from brackets: (0-0.5), (0.5-1), (1.2-1.5), (1.5-2)) .

Classifier performance as function of sample size of informative cases and fold increase in expression difference of informative probe sets ( fold changes are from brackets: (0-0.5), (0.5-1),(1-1.2), (1.2-1.5)) .

Classifier performance as function of informative feature size and overall feature size used for model building (fold=(0.5-1)).

Classifier performance as function of informative feature size and overall feature size used for model building (fold=(0-0.5)).

Classifier performance as function of fold increase in expression values of informative probe sets and sample size of informative cases (SpikedInFold= the med-points of the ranges)
